# Supplementary material for: Diversity and distribution of air-breathing sea slug genus Peronia Fleming, 1822 (Gastropoda: Onchidiidae) in southern Japanese waters
Source: PeerJ. 2022 Jul 19;10:e13720. doi: 10.7717/peerj.13720 (PMC9306565; doi:10.7717/peerj.13720)
Supplement: Supplemental Information 1 — Along with final species identification, specimen number, collection dates, size of live animals (Length/width mm) and GenBank accession numbers (first column: COI, second column: 16S). Previous sequences retrieved from GenBank are listed as specimen names “GB--”. [file peerj-10-13720-s001.docx]

| Species | Specimen number | Locality | Collection date | Animal size | COI | 16S |
| --- | --- | --- | --- | --- | --- | --- |
| ***P. verruculata* Unit #1** | SNB6 | Sunabe, Okinawa | 3 Aug 2020 | 50/-- mm | ON241923 | -- |
|  | NK3 | Nakagusuku, Okinawa | 19 Aug 2020 | 47/26 mm | ON241928 | -- |
|  | BAB0 | Baba Park, Okinawa | 16 Nov 2021 | 7/4 mm | ON241929 | -- |
|  | BAB8 | Baba Park, Okinawa | 4 Dec 2021 | 32/18 mm | ON241927 | ON242032 |
|  | AWS1 | Awase, Okinawa | 18 Aug 2020 | 28/9 mm | ON241849 | ON242030 |
|  | AWS2 | Awase, Okinawa | 18 Aug 2020 | 43/18 mm | ON241912 | ON242031 |
|  | AWS3 | Awase, Okinawa | 18 Aug 2020 | 20/12 mm | ON241850 | -- |
|  | OU1 | Oujima, Okinawa | 15 Sep 2020 | 70/37 mm | ON241926 | ON242045 |
|  | OU2 | Oujima, Okinawa | 15 Sep 2020 | 47/19 mm | ON241854 | ON242046 |
|  | OU3 | Oujima, Okinawa | 15 Sep 2020 | 30/18 mm | ON241855 | ON242047 |
|  | OU4 | Oujima, Okinawa | 15 Sep 2020 | 38/14 mm | ON241865 | ON242048 |
|  | GS1 | Gushikawa, Okinawa | 10 Aug 2020 | 50/39 mm | ON241867 | ON242033 |
|  | GS2 | Gushikawa, Okinawa | 10 Aug 2020 | 45/32 mm | ON241851 | ON242034 |
|  | GS3 | Gushikawa, Okinawa | 10 Aug 2020 | 41/32 mm | ON241911 | ON242035 |
|  | GS4 | Gushikawa, Okinawa | 10 Aug 2020 | 20/15 mm | ON241852 | ON242036 |
|  | GS5 | Gushikawa, Okinawa | 10 Aug 2020 | 40/14 mm | ON241853 | ON242037 |
|  | UKC1 | Ukachi, Okinawa | 29 Aug 2020 | 43/25 mm | ON241860 | ON242056 |
|  | UKC2 | Ukachi, Okinawa | 29 Aug 2020 | 43/25 mm | ON241861 | -- |
|  | UKC3 | Ukachi, Okinawa | 29 Aug 2020 | 34/12 mm | ON241862 | ON242057 |
|  | UKC4 | Ukachi, Okinawa | 29 Aug 2020 | 40/15 mm | ON241863 | ON243753 |
| ***P. verruculata* Unit #1** | SR1 | Seragaki, Okinawa | 6 Aug 2020 | 30/10 mm | ON241856 | ON243754 |
|  | SR2 | Seragaki, Okinawa | 6 Aug 2020 | 34/15 mm | ON241915 | ON243755 |
|  | SR3 | Seragaki, Okinawa | 6 Aug 2020 | 60/25 mm | ON241857 | ON241987 |
|  | SR4 | Seragaki, Okinawa | 6 Aug 2020 | 45/24 mm | ON241868 | ON243756 |
|  | SR5 | Seragaki, Okinawa | 6 Aug 2020 | 20/10 mm | ON241866 | ON243757 |
|  | TIM1 | Teima, Okinawa | 5 Nov 2020 | 45/20 mm | ON241859 | ON242053 |
|  | TIM2 | Teima, Okinawa | 5 Nov 2020 | 50/25 mm | ON241921 | ON241988 |
|  | TIM3 | Teima, Okinawa | 5 Nov 2020 | 19/13 mm | ON241858 | ON242054 |
|  | HD5 | Hedo, Okinawa | 5 Oct 2021 | 36/26 mm | ON241864 | ON242038 |
|  | HD7 | Hedo, Okinawa | 5 Oct 2021 | 31/26 mm | ON241922 | ON242039 |
|  | HD8 | Hedo, Okinawa | 5 Oct 2021 | 48/28 mm | ON241924 | ON241989 |
|  | HD12 | Hedo, Okinawa | 5 Oct 2021 | 30/20 mm | ON241925 | ON242040 |
|  | HD15 | Hedo, Okinawa | 5 Oct 2021 | 50/40 mm | ON241914 | ON242041 |
|  | IR1 | Iriomote Island | 15 Jul 2021 | 22/11 mm | ON241917 | ON242042 |
|  | IR2 | Iriomote Island | 15 Jul 2021 | 26/14 mm | ON241913 | ON242043 |
|  | IR3 | Iriomote Island | 15 Jul 2021 | 16/11 mm | ON241919 | ON242044 |
|  | IR4 | Iriomote Island | 15 Jul 2021 | 24/15 mm | ON241916 | ON242028 |
|  | IR5 | Iriomote Island | 15 Jul 2021 | 29/16 mm | ON241869 | ON242029 |
|  | TMR5 | Tomori, Amami Oshima | 4 Nov 2021 | 36/24 mm | ON241920 | ON242055 |
|  | KC1 | Nishidomari, Kochi | 18 Sep 2021 | 61/56 mm | ON241910 | ON241990 |
|  | KC2 | Nishidomari, Kochi | 18 Sep 2021 | 60/52 mm | ON241909 | ON241991 |
|  | GB1 | Ambon, Indonesia | 11 Feb 2014 | 35/25 mm | MT653153 | MT652698 |
| ***P. verruculata* Unit #1** | GB2 | Bali, Indonesia | 3 Apr 2010 | 20/15 mm | MT653156 | MT652701 |
|  | GB3 | Bohol, Philippines | 19 Jul 2014 | 40/25 mm | MT653186 | MT652731 |
|  | GB4 | Singapore | 1 Apr 2010 | 20/15 mm | MT653193 | MT652738 |
|  | GB5 | QLD, Australia | 20 Sep 2005 | 22/18 mm | MT653148 | MT652693 |
| ***P. verruculata* Unit #2** | GB6 | Andaman, India | 8 Jan 2011 | 25/15 mm | MT653200 | MT652745 |
|  | GB7 | Sumatra, Indonesia | 18 Oct 2012 | 25/20 mm | MT653206 | MT652751 |
| ***P. verruculata* Unit #3** | GB8 | Langkawi, Malaysia | 14 Jul 2011 | 35/30 mm | MT653212 | MT652757 |
|  | GB9 | Singapore | 29 Mar 2010 | 12/8 mm | MT653218 | MT652763 |
| ***P. verruculata* Unit #4** | GB10 | Western coast, India | 18 Dec 2011 | 55/30 mm | MT653220 | MT652765 |
|  | GB11 | Pakistan | Apr 2017 | 50/40 mm | MT653223 | MT652768 |
| ***P. verruculata* Unit #5** | GB12 | Madagascar | 16 May 2014 | 30/25 mm | MT653226 | MT652771 |
|  | GB13 | Mozambique | 2 Jul 2006 | 17/13 mm | HQ660045 | HQ659913 |
| ***P. peronii*** | SNB1 | Sunabe, Okinawa | 3 Aug 2020 | 110/100 mm | ON241889 | -- |
|  | SNB4 | Sunabe, Okinawa | 3 Aug 2020 | 90/-- mm | ON241890 | ON242016 |
|  | SNB5 | Sunabe, Okinawa | 3 Aug 2020 | 110/-- mm | ON241893 | ON242011 |
|  | NK1 | Nakagusuku, Okinawa | 19 Aug 2020 | 47/26 mm | ON241887 | ON242013 |
|  | NK2 | Nakagusuku, Okinawa | 19 Aug 2020 | 95/70 mm | ON241892 | -- |
|  | NK4 | Nakagusuku, Okinawa | 19 Aug 2020 | 45/25 mm | ON241888 | -- |
|  | HD1 | Hedo, Okinawa | 5 Oct 2021 | 105/81 mm | ON241891 | ON242015 |
|  | TMR6 | Tomori, Amami Oshima | 4 Nov 2021 | 90/60 mm | ON241886 | ON242014 |
|  | TMR7 | Tomori, Amami Oshima | 4 Nov 2021 | 80/60 mm | ON241894 | ON242012 |
|  | TMR13 | Tomori, Amami Oshima | 4 Nov 2021 | 14/8 mm | ON241895 | ON242017 |
| ***P. peronii*** | GB14 | Abu, Okinawa | July 2014 | -- | LC290402 | -- |
|  | GB15 | Guam | 27 Nov 2007 | 85/70 mm | MT653261 | MT652805 |
|  | GB16 | Madang, PNG | 27 Nov 2012 | 65/45 mm | MT653270 | MT652814 |
|  | GB17 | Madang, PNG | 12-14 Jun 2014 | 50/40 mm | MT653271 | MT652815 |
|  | GB18 | Mauritius | 11 Jun 2014 | 125/75 mm | MT653265 | MT652809 |
| ***P. okinawensis*** | SNB2 | Sunabe, Okinawa | 3 Aug 2020 | 65/-- mm | ON241896 | ON242020 |
|  | SNB3 | Sunabe, Okinawa | 3 Aug 2020 | 50/-- mm | ON241897 | ON242021 |
|  | HD2 | Hedo, Okinawa | 5 Oct 2021 | 60/35 mm | ON241898 | -- |
|  | HD3 | Hedo, Okinawa | 5 Oct 2021 | 60/40 mm | ON241899 | ON242018 |
|  | HD4 | Hedo, Okinawa | 5 Oct 2021 | 45/35 mm | ON241900 | -- |
|  | HD6 | Hedo, Okinawa | 5 Oct 2021 | 20/13 mm | ON241901 | ON242019 |
|  | HD9 | Hedo, Okinawa | 5 Oct 2021 | 28/18 mm | ON241905 | -- |
|  | TMR4 | Tomori, Amami Oshima | 4 Nov 2021 | 39/25 mm | ON241904 | ON242022 |
|  | TMR8 | Tomori, Amami Oshima | 4 Nov 2021 | 60/32 mm | ON241902 | ON242027 |
|  | TMR9 | Tomori, Amami Oshima | 4 Nov 2021 | 33/22 mm | ON241907 | ON242026 |
|  | TMR10 | Tomori, Amami Oshima | 4 Nov 2021 | 40/20 mm | ON241903 | ON242023 |
|  | TMR11 | Tomori, Amami Oshima | 4 Nov 2021 | 38/26 mm | ON241908 | ON242024 |
|  | TMR12 | Tomori, Amami Oshima | 4 Nov 2021 | 50/31 mm | ON241906 | ON242025 |
|  | GB19 | Hedo, Okinawa | 3 July 2004 | 20/17 mm | HQ660043 | HQ659911 |
|  | GB20 | Hedo, Okinawa | 3 July 2004 | 25/20 mm | MT653259 | MT652803 |
|  | GB21 | Hedo, Okinawa | 3 July 2004 | 27/25 mm | MT653260 | MT652804 |
| ***P. setoensis*** | BAB1 | Baba Park, Okinawa | 16 Nov 2021 | 8/6 mm | ON241872 | ON241995 |
| ***P. setoensis*** | BAB2 | Baba Park, Okinawa | 16 Nov 2021 | 10/7 mm | ON241878 | ON241996 |
|  | BAB3 | Baba Park, Okinawa | 16 Nov 2021 | 10/7 mm | -- | -- |
|  | BAB4 | Baba Park, Okinawa | 16 Nov 2021 | 7/4 mm | ON241875 | ON241997 |
|  | BAB5 | Baba Park, Okinawa | 16 Nov 2021 | 5/4 mm | ON241874 | ON241998 |
|  | BAB6 | Baba Park, Okinawa | 16 Nov 2021 | 14/5 mm | -- | ON241999 |
|  | BAB7 | Baba Park, Okinawa | 16 Nov 2021 | 8/7 mm | ON241880 | ON242000 |
|  | BAB9 | Baba Park, Okinawa | 16 Nov 2021 | 6/4 mm | ON241877 | ON242001 |
|  | BAB10 | Baba Park, Okinawa | 16 Nov 2021 | 16/11 mm | ON241885 | ON242010 |
|  | BAB11 | Baba Park, Okinawa | 16 Nov 2021 | 7/4 mm | ON241879 | ON242002 |
|  | BAB12 | Baba Park, Okinawa | 16 Nov 2021 | 15/9 mm | ON243660 | ON242003 |
|  | BAB13 | Baba Park, Okinawa | 16 Nov 2021 | 15/8 mm | ON241873 | ON242004 |
|  | BAB14 | Baba Park, Okinawa | 16 Nov 2021 | 13/9 mm | ON241876 | ON242005 |
|  | HD10 | Hedo, Okinawa | 5 Oct 2021 | 20/11 mm | ON241883 | ON242006 |
|  | HD11 | Hedo, Okinawa | 5 Oct 2021 | 20/13 mm | ON241881 | ON242007 |
|  | HD13 | Hedo, Okinawa | 5 Oct 2021 | 20/13 mm | ON241882 | ON242008 |
|  | HD16 | Hedo, Okinawa | 5 Oct 2021 | 20/14 mm | ON241884 | ON242009 |
|  | TMR1 | Tomori, Amami Oshima | 4 Nov 2021 | 24/21 mm | ON241871 | ON241992 |
|  | TMR2 | Tomori, Amami Oshima | 4 Nov 2021 | 27/20 mm | ON241870 | ON241993 |
|  | TMR14 | Tomori, Amami Oshima | 4 Nov 2021 | 28/16 mm | ON243661 | ON241994 |
|  | GB22 | Nishimuro, Wakayama | 2014 | 15/10 mm | MT653279 | MT652823 |
|  | GB23 | Nishimuro, Wakayama | 2014 | 15/10 mm | MT653280 | MT652824 |
|  | GB24 | Nishimuro, Wakayama | 30 Aug 2014 | 13/8 mm | MT653281 | MT652825 |
| ***P. setoensis*** | GB25 | Nishimuro, Wakayama | 30 Aug 2014 | 20/15 mm | MT653282 | MT652826 |
|  | GB26 | Nishimuro, Wakayama | 30 Aug 2014 | 10/5 mm | MT653283 | MT652827 |
|  | GB27 | Nishimuro, Wakayama | 30 Aug 2014 | 12/10 nm | MT653284 | MT652828 |
| ***Wallaconchis ater*** | GB28 | Bohol, Philippines | -- | -- | MG970830 | MG970915 |
|  | GB29 | Halmahera, Indonesia | -- | -- | MG970836 | MG970916 |
