# Supplementary material for: Diversity and distribution of air-breathing sea slug genus Peronia Fleming, 1822 (Gastropoda: Onchidiidae) in southern Japanese waters
Source: PeerJ. 2022 Jul 19;10:e13720. doi: 10.7717/peerj.13720 (PMC9306565; doi:10.7717/peerj.13720)
Supplement: Supplemental Information 2 [file peerj-10-13720-s002.docx]

| **Species** | **Haplotype** | **SNB** | **BAB** | **NK** | **AWS** | **OU** | **GSK** | **UKC** | **SR** | **TIM** | **HD** | **IR** | **TMR** | **KC** |
| --- | --- | --- | --- | --- | --- | --- | --- | --- | --- | --- | --- | --- | --- | --- |
| ***P. verrucualata*** | 1 | 1 | 2 |  | 2 | 2 | 2 | 3 | 1 | 2 | 2 |  |  |  |
|  | 2 |  |  |  |  |  |  |  | 1 |  |  |  |  |  |
|  | 3 |  |  |  |  | 1 |  |  |  |  |  |  |  |  |
|  | 4 |  |  |  |  |  |  |  |  |  | 1 |  |  |  |
|  | 5 |  |  | 1 |  |  |  |  |  |  |  |  |  |  |
|  | 6 |  |  |  |  |  |  |  |  |  | 1 |  |  |  |
|  | 7 |  |  |  |  | 1 |  |  |  |  |  |  |  |  |
|  | 8 |  |  |  |  |  |  |  |  | 1 |  |  | 1 |  |
|  | 9 |  |  |  |  |  |  |  |  |  |  |  |  | 1 |
|  | 10 |  |  |  |  |  |  |  |  |  |  |  |  | 1 |
|  | 11 |  |  |  | 1 |  | 1 |  |  |  |  |  |  |  |
|  | 12 |  |  |  |  |  |  |  |  |  | 1 | 2 |  |  |
|  | 13 |  |  |  |  |  |  |  | 1 |  |  |  |  |  |
|  | 14 |  |  |  |  |  |  |  |  |  |  | 1 |  |  |
|  | 15 |  |  |  |  |  |  |  |  |  |  | 1 |  |  |
|  | 16 |  |  |  |  |  | 1 |  | 1 |  |  |  |  |  |
|  | 17 |  |  |  |  |  |  |  |  |  |  | 1 |  |  |
| ***P. setoensis*** | 18 |  | 8 |  |  |  |  |  |  |  |  |  |  |  |
|  | 19 |  | 2 |  |  |  |  |  |  |  |  |  |  |  |
|  | 20 |  |  |  |  |  |  |  |  |  | 2 |  |  |  |
|  | 21 |  |  |  |  |  |  |  |  |  | 2 |  |  |  |
|  | 22 |  | 1 |  |  |  |  |  |  |  |  |  |  |  |
|  | 23 |  |  |  |  |  |  |  |  |  |  |  | 3 |  |
| ***P. peronii*** | 24 | 2 |  | 1 |  |  |  |  |  |  | 1 |  | 1 |  |
|  | 25 |  |  |  |  |  |  |  |  |  |  |  | 1 |  |
|  | 26 |  |  | 1 |  |  |  |  |  |  |  |  |  |  |
|  | 27 |  |  |  |  |  |  |  |  |  |  |  | 1 |  |
|  | 28 |  |  | 1 |  |  |  |  |  |  |  |  |  |  |
|  | 29 | 1 |  |  |  |  |  |  |  |  |  |  |  |  |
| ***P. okinawensis*** | 30 | 2 |  |  |  |  |  |  |  |  | 4 |  | 3 |  |
|  | 31 |  |  |  |  |  |  |  |  |  | 1 |  |  |  |
|  | 32 |  |  |  |  |  |  |  |  |  |  |  | 1 |  |
|  | 33 |  |  |  |  |  |  |  |  |  |  |  | 1 |  |
|  | 34 |  |  |  |  |  |  |  |  |  |  |  | 1 |  |
