# Supplementary figures and images for: Diversity and distribution of air-breathing sea slug genus Peronia Fleming, 1822 (Gastropoda: Onchidiidae) in southern Japanese waters
Source: PeerJ. 2022 Jul 19;10:e13720. doi: 10.7717/peerj.13720 (PMC9306565; doi:10.7717/peerj.13720)

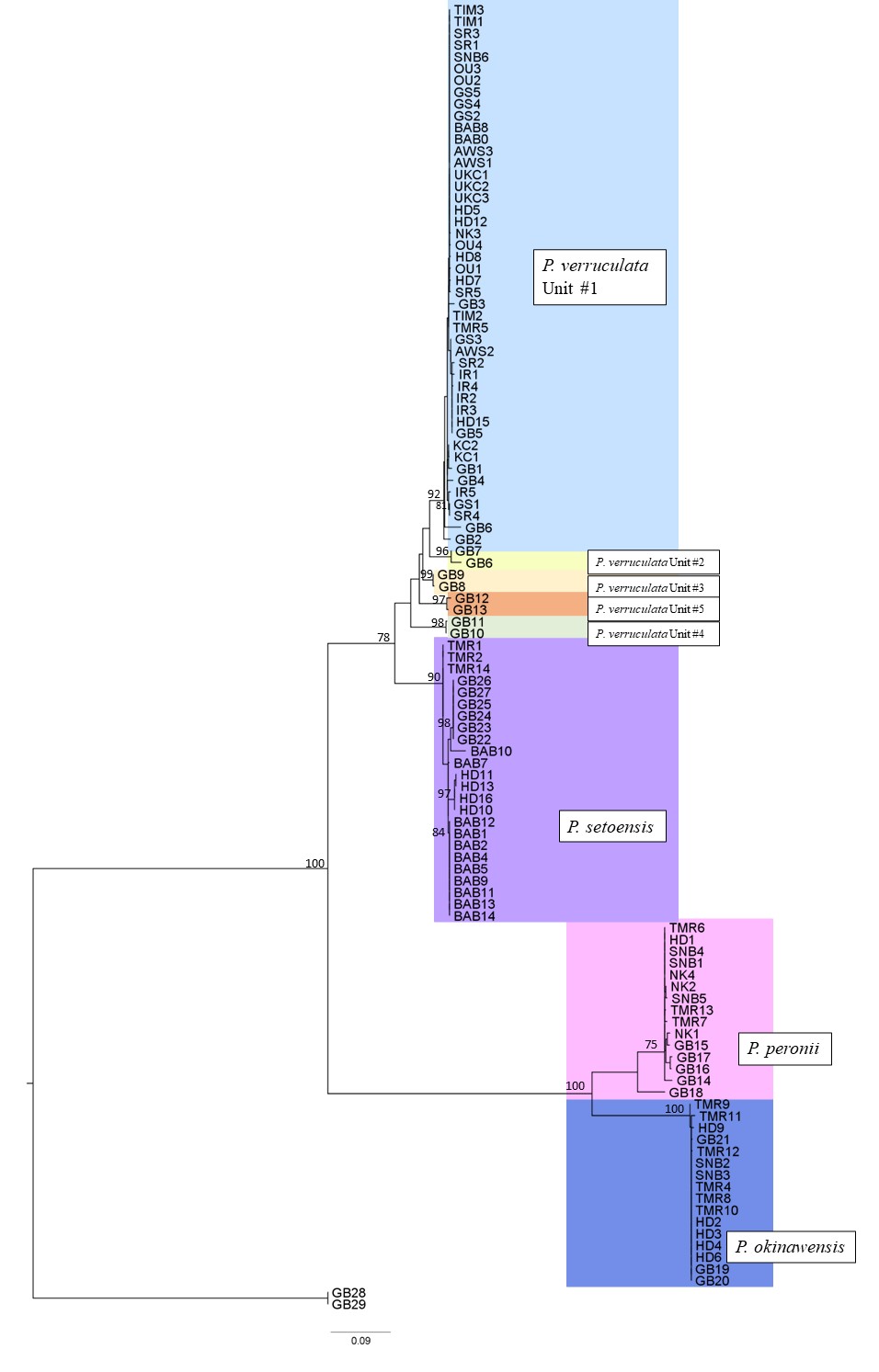

Supplement: Supplemental Information 4 — Bootstrap values (≧75%) are listed at each node. [file peerj-10-13720-s004.jpg]

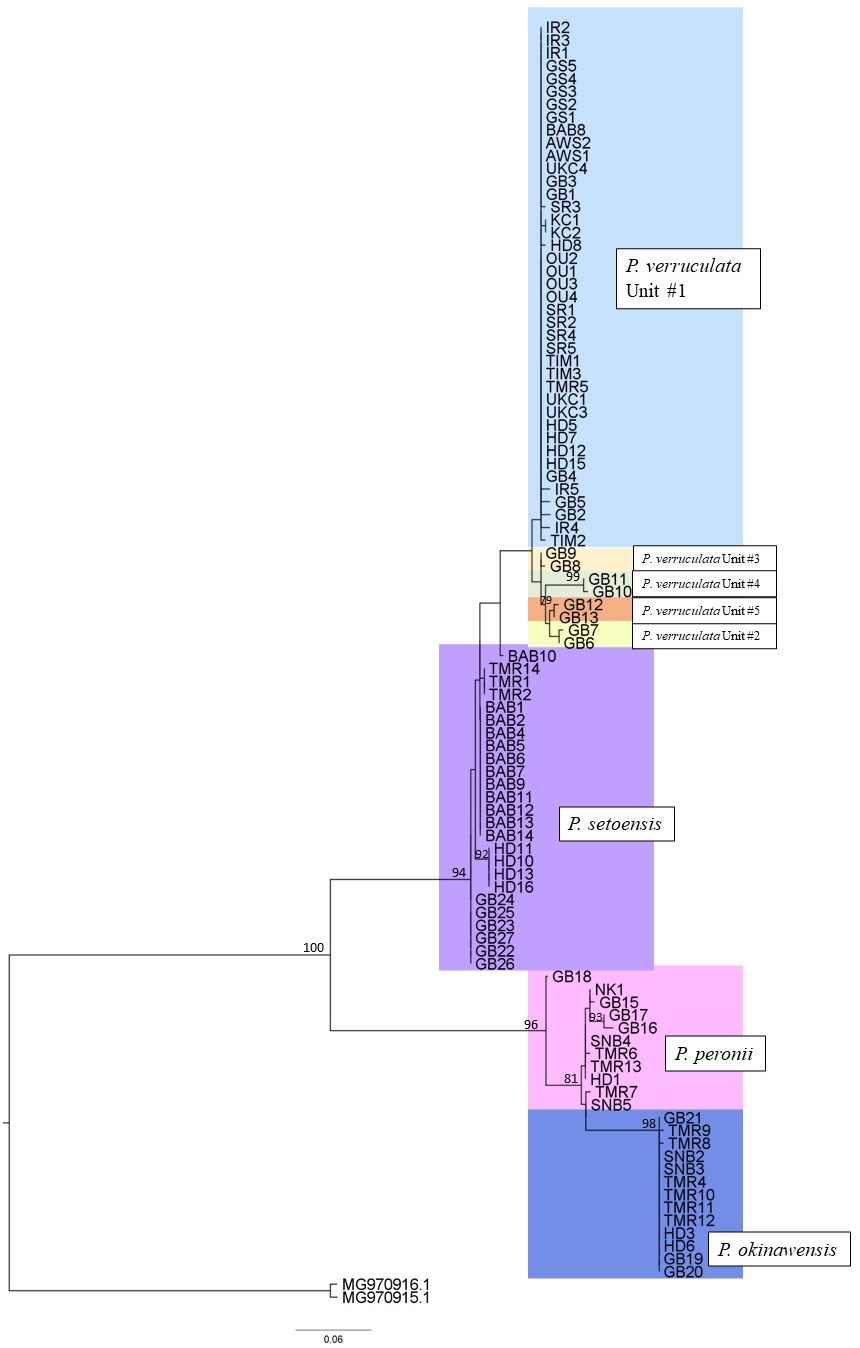

Supplement: Supplemental Information 5 — Bootstrap values (≧75%) are listed at each node. [file peerj-10-13720-s005.jpg]
